# Mitochondrial Diversity and Phylogenetic Relationship of Eight Native Bulgarian Sheep Breeds

**DOI:** 10.3390/ani13233655

**Published:** 2023-11-25

**Authors:** Georgi Kalaydzhiev, Nadezhda Palova, Heliana Dundarova, Lyudmila Lozanova, Ivan Mehandjyiski, Georgi Radoslavov, Peter Hristov

**Affiliations:** 1 Department Livestock—Ruminants and Technologies of Animal Products, Trakia University, 6000 Stara Zagora, Bulgaria; gopo@abv.bg; 2 Scientific Center of Agriculture, Agricultural Academy, 8300 Sredets, Bulgaria; nadejda_palova@abv.bg; 3 Institute of Biodiversity and Ecosystem Research, Bulgarian Academy of Sciences, 1113 Sofia, Bulgaria; h.vulgaris@gmail.com (H.D.); lusilozanova@gmail.com (L.L.); gradoslavov@gmail.com (G.R.); 4 Research Center of Stockbreeding and Agriculture, Agricultural Academy Bulgaria, 4700 Smolyan, Bulgaria; bg_porodi07@abv.bg

**Keywords:** mitochondrial DNA, D-loop region, genetic diversity, phylogeny

## Abstract

**Simple Summary:**

Mitochondrial DNA (mtDNA) is one of the most often used genetic markers in animal population studies. Its relevance as a genetic marker is due to the high mutation rate and variability, the maternal inheritance, and the supposedly nearly neutral mode of evolution. Therefore, mtDNA diversity is typically assumed to reflect demographic effects, i.e., variations in population size between species or populations, which makes it a popular tool for conservation purposes. Thus, mtDNA diversity allows a better understanding of the evolutionary plasticity and provides insights into the molecular and evolutionary mechanisms modeling mitochondrial genomes.

**Abstract:**

The geographical, geomorphological, and climatic characteristics of Bulgaria are particularly favorable for animal breeding and, above all, for pastoral farming and sheep breeding. These conditions created prerequisites for the creation of about 30 unique local breeds of sheep. In this study we investigated the genetic diversity of eight of the most popular Bulgarian native breeds, based on the sequence analysis of a part of the mitochondrial D-loop region. An almost entire mitochondrial DNA (mtDNA) D-loop region (1180 bp) was amplified and sequenced. The obtained results showed the presence of a large number of haplotypes–225, belonging to two main haplogroups. The majority of samples showed a high prevalence of the European haplogroup B (95.2%) while the remaining individuals were assigned to haplogroup A (4.8%). None of the other reported mitochondrial haplogroups were observed. The number of polymorphic sites, nucleotide and haplotype diversity was high (240, 0.01237, and 0.9968, respectively), which is evidence for multiple maternal origins in all populations. The Tajima D-test value in all the study populations was −1.905 (*p* < 0.05), indicating that the abundance of rare alleles was most likely due to population expansion after a recent bottleneck. The Median joining network showed that almost all haplotypes belonging to haplogroup B formed a star-like network, which revealed a weak genetic differentiation and a large gene flow between the Bulgarian native breeds.

## 1. Introduction

The domestic sheep (*Ovis aries*) is one of the livestock species whose breeding activity has proceeded over the centuries mainly in several directions—wool, dairy, meat, and hide production. According to archeological and historical evidence and recent molecular genetics data, the sheep (*Ovis aries*) was domesticated near the Fertile Crescent, approximately 10,500 years ago [1,2]. It is supposed that the predecessor of the domestic sheep is the Asiatic mouflon (*Ovis orientalis*), also known as *O. gmelinii* [3,4]. It was initially believed that the European mouflon (*O. musimon*) could be the ancestor of the domestic sheep, based on archeological and genetic evidence as well as the same chromosome number as that of domestic sheep (2n = 54) [5,6]. It was considered that during the Neolithic transition, the early Neolithic farmers introduced the already domesticated sheep initially into the Mediterranean part of Cyprus ~ 10,000 BC, and after that to Corsica and Sardinia ~ 6–7000 BC [7,8], where the feral population of European mouflon appeared [9].

The dispersal of early domesticated sheep into Neolithic Europe is inextricably linked to the emergence of different archeological entities (i.e., “cultures”) in ancient civilizations [10,11]. The first early farmers settled in the Balkan Peninsula and set the beginning of the Karanovo I/II-Koros-Starcevo culture [12,13]. According to archaeological evidence, the farmers inhabiting the Balkans additionally spread throughout Europe in at least two directions—the first route was initiated approximately 5900 years BC and formed a different culture, the Impressa, located sporadically along the Mediterranean coast [14,15]. When the human expansion reached the Iberian Peninsula ca 5500 BC, a new culture, named Cardial, emerged [16]. The second neolithization of Europe occurred in parallel with the Cardial culture reaching large parts of Central Europe. This expansion was realized via a mainland route along the Danube River into Central Europe represented by the LBK (Linearbandkeramik) tradition [17,18]. LBK culture known as Danubian Culture expanded over large areas of Europe north and west of the Danube River around 5000 BC [19].

All these observations suggest that the neolithization of prehistoric Europe, first via the Mediterranean coastal route and after about 500 years via the Danube route, and the formation of the earliest European cultures inevitably had an impact on the genetic diversity of domestic sheep due to human-mediated migrations into Neolithic Europe.

The sequence analysis of the complete mitochondrial genome, the mtDNA D-loop fragment and the *cytB* gene of modern sheep (from a different geographical area) has shown the presence of five major mitochondrial haplogroups—A, B, C, D, and E [20,21,22]. Haplogroups A and B are with the highest frequencies among modern breeds, although they are found in wild and ancient sheep worldwide with different frequencies [23,24,25]. Currently, haplogroup A dominates in domestic sheep and is specific to modern Asiatic sheep breeds [23,26,27], while haplogroup B is considered typical for European sheep breeds [21,28].

Haplogroup C is the third haplogroup with high distribution among modern and ancient sheep, especially in the Middle East [23,29,30]. Haplogroup C is established most often in domestic sheep from Turkey but has also been identified in sheep from the Iberian Peninsula [31], the southern countries of the Balkan Peninsula (Albania and Greece) [32], China [33], the North Caucasus (Stavropol region) [34], and many regions of Asia [35].

The last two haplogroups, D and E, have been observed with very low frequencies among both modern and ancient sheep samples [23,34,36]. Haplogroup D has been identified only in modern domestic sheep from the Caucasus [34], while haplogroup E has been observed both in modern and ancient samples in the Middle East [36].

The diverse ecological and economic conditions in Bulgaria and the various needs and interests of the local people in our country allowed raising a large number of native sheep breeds. According to official statistics, there are currently 18 native sheep breeds in Bulgaria [37]. According to the total population size, Bulgarian native sheep breeds can be classified in several categories of risk status using FAO criteria [38]. Six of the native sheep breeds are “endangered” and five breeds are “vulnerable”. The remaining seven breeds have a “not at risk” status.

To date, the majority of studies on the genetic diversity among native Bulgarian sheep have been performed only on autosomal short tandem repeat variations (STR, microsatellites) [39,40,41,42,43,44]. In this research, carried out in 2023, we evaluated for the first time the genetic diversity of eight native Bulgarian sheep breeds which are threatened with extinction, based on the sequence polymorphism of the mitochondrial control region (D-loop region). We also performed a phylogenetic and phylogeographical analysis to test the relationship between our samples and other European sheep breeds from the Balkan countries. In order to fill the evolutionary gaps between the native Bulgarian sheep and other sheep breeds from different European countries, we used the available in Gen Bank sequences of mitochondrial control region.

## 2. Materials and Methods

### 2.1. Animal Welfare and Ethical Statement

All experimental procedures were reviewed and approved by the Animal Research Ethics Committee of the Bulgarian Food Safety Agency (BFSA) (Art. 154 of the Law on Veterinary Activity), in accordance with European Union Directive 86/609.

### 2.2. Sample Collection

**Figure 1:**
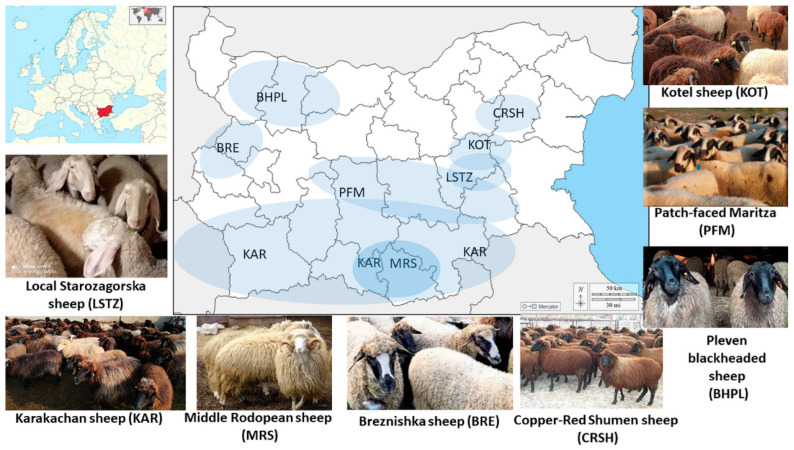
Geographical distribution of eight Bulgarian sheep breeds analyzed in this study. Please note that the proportions and distances of the map are not displayed realistically, they are only for orientation.

Blood samples were collected by using a 6 mL K_2_EDTA BD vacutainer^®^ (Becton Dickinson, Franklin Lakes, NJ, USA) from 310 unrelated sheep belonging to 8 native Bulgarian breeds: Breznishka (BRE, n = 17; 1 flock), Karakachan (KAR, n = 60; 2 flocks), Kotel (KOT, n = 36; 2 flocks), Pleven Blackhead (BHPL, n = 32; 1 flock), Copper-Red Shumen sheep (CRSH, n = 34; 1 flock), Middle Rhodopean Sheep (MRS, n = 48; 2 flocks), Local Starozagorska (LSTZ, n = 69; 3 flocks), and Patch-faced Maritza (PFM, n = 14; 1 flock) (Figure 1). All sampled animals belonged to pure native Bulgarian breeds and were selected with their respective breeders association to ensure that they were not closely related. In addition, the pedigree lists were obtained from records in the Executive Agency for Selection and Reproduction in Animal Husbandry.

### 2.3. DNA Extraction, PCR Amplification, and Sequencing

DNA was isolated from all blood, using a commercial GeneMATRIX Quick Blood DNA Purification Kit (EUR_X_ Sp. z o.o., Gdańsk, Poland) in accordance with the manufacturer’s guidelines. The integrity of the extracted DNA was checked by 1% agarose gel electrophoresis and then visualized on UV transilluminator gel documentation systems after staining with SimpliSafe™ (Cat. No. E4600; EURx Ltd., Gdansk, Poland). The isolated DNA samples were preserved at −20 °C until further analysis.

A fragment of 1428 bp (from 15,346 bp to 157 bp, ovine mitochondrial DNA (mtDNA) reference sequence NC_001941) [45] of the mtDNA D-loop region was amplified using the following primers: 15346FOR (5′-GGAGAACAACCAACCTCCCTA-3′) and 157REV (5′-TGATTCGAAGGGCGTTACTC-3′) [46].

The PCR mixtures contained 25 µL of Color Taq PCR Master Mix (2×) (Cat. No. E2525, EURx Ltd., Gdansk, Poland), 1 µM of each primer (FOR/REV), and 1 µL of extracted DNA for a total volume of 50 µL. All PCR amplifications were carried out using a LifeExpress Classic Thermal Cycler (BIOER Technology Co., Ltd., Kampenhout, Belgium) under the following conditions: initial denaturation at 94 °C for 5 min; 30 cycles (denaturation at 94 °C for 30 s; primer annealing at 50 °C for 30 s; extension at 72 °C for 1.30 min), and final extension at 72 °C for 10 min.

The successfully amplified PCR products were visualized on 1% agarose gel stained with SimplySafe™ (Cat. No. E4600, EURx Ltd., Gdansk, Poland). The fragment size was determined using DNA Molecular Weight Marker XIV (Cat. No. 11721933001, Merck Bulgaria EAD, Sofia, Bulgaria). The PCR products were purified by a PCR/DNA Clean-Up Purification Kit (Cat. No. E3520, EURx Ltd., Gdansk, Poland) and sequenced in both directions by a PlateSeq kit (Eurofins Genomics Ebersberg, Germany).

### 2.4. Bioinformatics and Data Analysis

All 310 obtained DNA sequences (about 1180 bp) were manually edited and aligned with an *Ovis aries* reference sequence of haplogroup B (NC_001941) [45] using the MUSCLE algorithm [47] as implemented in the MEGA v. 11 software [48] and have been deposited in GenBank with the accession numbers OR137208-OR137517.

For each breed, DnaSP 5.0 [49] was used to determine the number of haplotypes (h), nucleotide diversity (π), Haplotype diversity, (Hd), average number of nucleotide differences (Kt), number of segregating sites (S), and nucleotide diversity with JC (PiJC). DnaSP 5.0 was also used to calculate neutrality indices (Tajima’s D, and Fu’s D and F tests values) [50,51] and to estimate mismatch distribution.

Principal component analyses (PCA) were performed using Excel software Version 2206 implemented by XLSTAT [52]. Two PCA coordinates were carried out: one by considering only the sample described in the present study; the other one including the large database of mtDNA control region sequences available in GenBank: MW427961-MW428078 [21]; JN184789-JN184999 [32]; DQ851886DQ8552279 [36]; KT158312-KT158460 [53]; OR459774-OR459640 [54]; EU019139- EU019189 [55]; DQ491576–DQ491736 [56]. These datasets were obtained from different European regions with the aim to compare the genetic similarity or distance between our samples and those from other European locations.

For haplogroup identification, the MitoToolPy software (http://www.mitotool.org/ accessed on 22 February 2023) was used [57].

All analyzed sequences were exported from DnaSP 5.0 to PopArt v1.7 [58] to construct the haplotype network. The geographic distribution of mtDNA haplotypes was visualized by producing a haplotype network, using the Median-joining option of the program PopArt v1.7.

The analysis of the molecular variance (AMOVA) [59], implemented in Arlequin v 3.5.2.2 (http://cmpg.unibe.ch/software/arlequin35/Arl35Downloads.html accessed on 20 August 2023) [59], was used to assess the population differentiation and to evaluate the genetic variability among and within populations.

Neighbour-joining (NJ) tree using MEGA6 [60] was constructed for exploring the association between the haplotypes of all sheep breeds with bootstrap 1000 replications. The evolutionary divergence between the studied breeds was conducted using the Maximum Composite Likelihood model [60]. The analysis involved 310 nucleotide sequences. All ambiguous positions were removed for each sequence pair (pairwise deletion option). There were a total of 1180 positions in the final dataset. Evolutionary analyses were conducted in MEGA11 [48].

## 3. Results

### 3.1. mtDNA Sequence Polymorphis in Native Bulgarian Sheep Breeds

The 1180 bp long region of the mitochondrial D-loop obtained from 310 unrelated native Bulgarian sheep revealed 225 different haplotypes with 240 polymorphic sites. The overall values for haplotype and nucleotide diversities were 0.9968 and 0.01237, respectively, for all eight studied breeds (Table 1). Among the eight Bulgarian breeds, Kotel sheep and Patch-faced Maritza showed the highest values of genetic diversity in terms of haplotype (1.000 and 0.995, respectively) and nucleotide (0.017 and 0.016, respectively) diversity. The lowest values of these indices were observed for Breznishka and Middle Rhodopean sheep (*H*_d_ = 0.912, π = 0.009; *H*_d_ = 0.953, π = 0.009, respectively). Pleven Blackhead and Copper-Red Shumen sheep were completely similar in terms of haplotype (0.986) and nucleotide (0.011) diversity (Table 1).

The Tajima’s D test was negative for all populations but statistically not significant in most of them, except for Karakachan and Kotel sheep, indicating an excess of rare polymorphic nucleotide sites (Table 1). The results of the Fu and Li’s D and F tests, associated with the distribution of haplotypes, showed negative values in all the studied native Bulgarian breeds. This suggests an excess of rare haplotypes, which is confirmed by the large number of haplotypes observed in our study—225.

Generally, the negative values of the Tajima’s D test as well as the Fu and Li’s D and F tests indicate the presence of an excess of rare mutations, but this excess is not statistically significant (Table 1).

### 3.2. Haplotype Distribution among Breeds, Haplogroup Identification and Haplotype Network

In all native Bulgarian sheep breeds, a total of 225 different haplotypes were observed (Table 1). The highest number of haplotypes was identified for Local Starozagorska and Karakachan breeds (54 and 40, receptively), while Breznishka and Patch-faced Maritza sheep showed the lowest number (11 and 13, respectively), probably due to the small number of samples from these breeds.

The distribution of haplotypes of the mitochondrial D-loop region among all sheep breeds is presented in Table S1. There were 15 haplotypes, each of which was shared by at least two different breeds, e.g., Hap9 was present in three samples—one from Breznishka sheep and two from Local Starozagorska sheep; Hap20 in four samples—one from Breznishka sheep and three from Karakachan sheep; Hap80—in two samples from Kotel sheep and two from Copper-Red Shumen sheep, etc. The remaining haplotypes were specific to the given studied breed.

All identified haplotypes were assigned in only two haplogroups (A and B), using the MitoToolPy program. Haplogroup B was with the highest prevalence among the samples, showing a frequency of 95.2%. This haplogroup was present at an extremely high frequency in all sheep breeds (Table S1). In comparison, haplogroup A was present in only 15 out of 310 individuals. Moreover, haplogroup A was not observed in all sheep breeds. Sequence analysis showed that in some breeds such as Patch-faced Maritza and Kotel sheep, this group showed a frequency of about 14% (Table S1).

**Figure 2:**
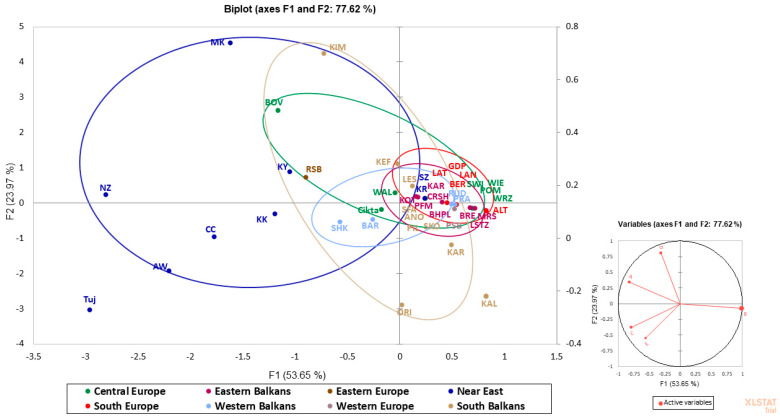
A two—dimensional PCA plot generated by available European and Middle East sheep breeds mtDNA data (Table S1). The macrogeographic labels, indicated in bold, represent the centroids of breeds from the area. On the right is the plot of the contribution of each haplogroup (A–E) to the first and the second PCA axes of the original variables.

In order to graphically display the mitochondrial relationships among the analyzed sheep breeds as well as the haplogroup frequencies in different European populations mainly from the Balkans, we performed a principal component analysis (PCA)—a method that considers each haplogroup as a discrete variable and allows a summary of the initial dataset into principal components (PCs) (Figure 2). The PCA analysis revealed the close localization of Bulgarian native sheep breeds with other European breeds. This is not surprising, considering the high frequency of haplogroup B in all European sheep populations. The distance between European sheep breeds and those from the Near East is clearly visible from the PCA plot. The main reason for this is the observed haplogroup C with a different frequency in Near Eastern sheep breeds (Table S1). While the presence of haplogroup C in some of the Russian native sheep breeds is legitimate as part of Asian breeds, in sheep breeds from the Western Balkans (Albania), haplogroup C is also present, which is the reason for the separation of the sheep breeds Bardhoka and Shkordane from the main cluster.

### 3.3. Genetic Differentiation between Bulgarian Native Sheep Breeds

We also examined the genetic distance within and among the studied native Bulgarian breeds, measured in nucleotide substitutions per site. The obtained results clearly showed the very low genetic differentiation between them, thus supporting the evidence from the network and PCA analysis (Table 2). The lowest genetic distance was observed between Breznishka and Karakachan sheep, while Kotel and Patch-faced Maritza showed the highest genetic differentiation.

In addition, we calculate the FST value between each two breeds (Table S2). The highest genetic differentiation was observed between BRE and MRS sheep breeds (0.064), while the lowest was found between LSTZ and PFM (0.003).

The AMOVA analysis at the breed’s level supported low genetic differentiation among studied breeds (Table 3). The obtained results showed a very low genetic distinction between the studied eight sheep populations, with only 2.42% variation among the groups and 97.58% within them.

**Figure 3:**
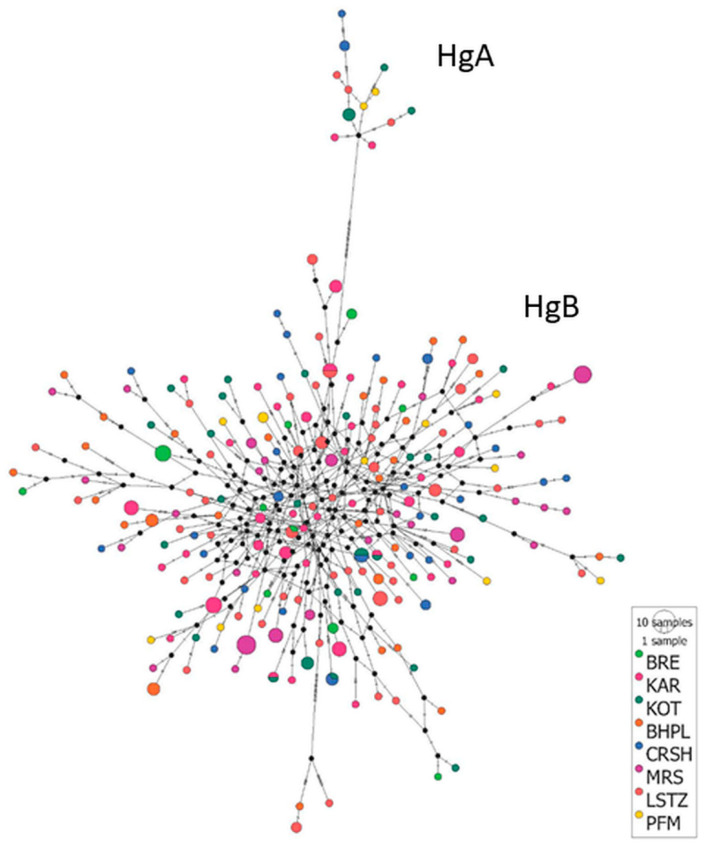
Median joining network of D-loop mitochondrial sequences constructed with PopArt v1.7. Haplotypes are represented by circles whose sizes are proportional to the number of individuals. Different colors represent different sheep breeds. The abbreviations of Bulgarian sheep populations are provided in Table S1.

The low genetic differentiation between the breeds is visualized in Figure 3. The constructed median-joining network showed that almost all sequences were clustered into haplogroup B, with a total of 213 distinct haplotypes. The remaining 12 haplotypes assigned to haplogroup A were separated away from the center of the diagram. In haplogroup A, the breeds are mainly concentrated in plain regions of Southeastern Bulgaria (Thracian lowland), while haplogroup B include breeds mainly disseminated in the mountainous regions of the country. Thus, the PCA plot represented haplotype clustering into two main haplogroups, A and B, with many individuals that mixed without a clear distinction between individual breeds. The haplotype network analysis showed a star-like structure for haplogroup B, suggesting population expansion, which confirmed the results obtained from the Tajima’s D test (Table 1).

**Figure 4:**
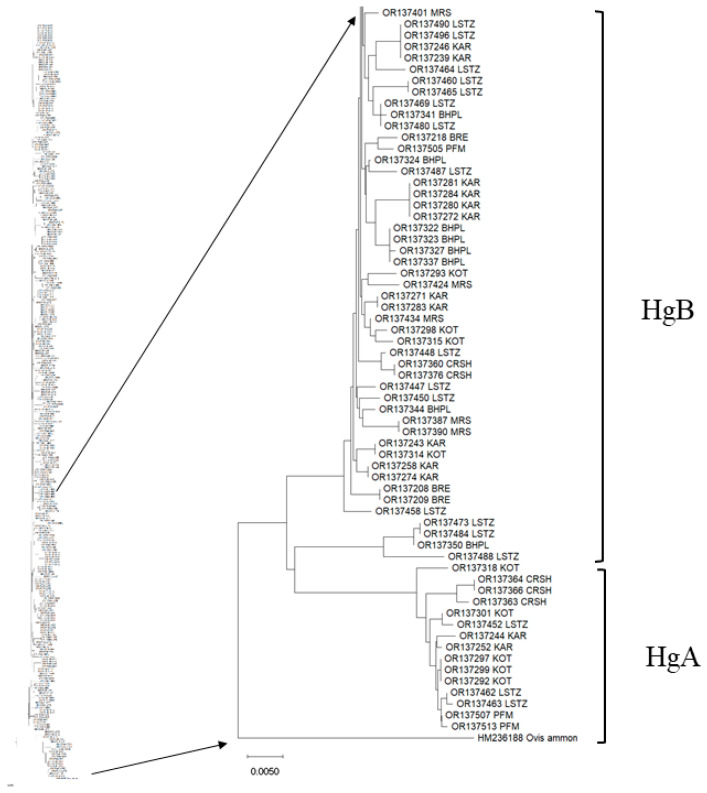
Neighbour-joining haplotype tree constructed from Kimura two-parameter distance of the 213 distinct haplotypes of the eight native Bulgarian sheep breeds. Bootstraps of 1000 replicates were used to test the robustness and the tree rooted by using the argali (*O. ammon*) D-loop sequence (GenBank Acc. No. HM236188). The abbreviations of Bulgarian sheep populations are provided in Table S1.

Genetic relationship among all sheep breeds was further confirmed by the construction of NJ phylogenetic tree (Figure 4). The results support information obtained from PCA and Median joining network analyses. The majority of animals (n = 295) grouped into the previously described haplogroup B. Haplogroup A was the most prevalent in Kotel sheep breed (n = 5), Copper-Red Shumen sheep breed (n = 3), Local Starozagorska sheep breed (n = 3) etc.

## 4. Discussion

### 4.1. mtDNA Diversity in Bulgarian Native Sheep Breeds

The intensification of the output of animal production in Bulgaria during the last decades has led to a decrease in the number of breeds used as the basis for production. Breeds having a lower total productivity or not suitable for keeping in the conditions of industrial complexes were quickly replaced by more productive and commercial breeds through crossbreeding [61,62]. Together with the loss of genes caused by the extinction of breeds, there has been some loss of genes because of the intensive selection that has been practiced within breeds. This process, characteristic of all countries with developed animal breeding, is very clearly apparent in the countries having large socialistic farms where animals are under industrial conditions of exploitation [63,64].

In this study, we evaluated the mitochondrial diversity (1180 bp of D-loop region) of eight native Bulgarian native breeds. The high prevalence of haplogroup B in about 95% of our samples is not unusual as it is widespread in European sheep breeds [21,28]. The high frequency of this haplogroup suggests much older population dissemination from the domesticated center than the other haplogroups. The complete dominance of haplogroup B in Europe also may indicate the expansion of the European population from only a few individuals carrying haplogroup B, i.e., bottleneck effect [30].

The Asian specific haplogroup A was present at a very low frequency in our samples (~5.0%). A relatively high frequency of this haplogroup was observed in Kotel and Patch-faced Maritza sheep (~14%) (Table S1). These breeds are distributed in different regions of the country and are not really related to each other. The spread of haplogroup A in Europe is assumed to have occurred through the so-called Secondary Products Revolution [65,66]. This innovation in the Old World occurred during the Middle Chalcolithic (around 4th millennium BC) [67,68]. This period marked a new world revolution of human-animal relationships, i.e., from the initial use of domestic animals for meat production to the use of their secondary products, such as milk, wool, hide, and new applications, such as traction power or transport [69,70]. Previous researches in the countries of the Balkan Peninsula revealed the presence of haplogroup A, with a different frequency, in sheep from the South Balkans—Greece (~8%) [32], the Western Balkans—Bosnia and Herzegovina, and Albania [32,55] (7.6% and 6.3%, respectively). These results indicate that haplogroup A is present at a low frequency among Balkan sheep breeds. Conversely, the frequency of haplogroup A in Iberian sheep is quite high (19%) [56]. Haplogroup A appears to be of high frequency in Merino sheep breeds because there is a “cradle” of Merino sheep populations on the Iberian Peninsula [56,71]. It is assumed that specialization for wool production occurred in the early Iron Age (1200 years BCE) [72], replacing, in many areas, the primitive sheep populations [73]. The so called “Merino Breed” industry spread in many countries worldwide [3,74]. The latter is confirmed by the presence of haplogroup A, with different frequencies, in many Merino breeds from Central Europe (Austria, Slovenia, Hungary, and Poland) [21,53], South Balkans (Greece) [32], Western Balkans (Bosnia and Herzegovina, Albania) [55,56], etc.

Surprisingly, we did not detect haplogroup C in our set, although this haplogroup has been found in some sheep breeds from the Balkan Peninsula—Bosnia and Herzegovina and Albania [55,56]. The fact that this haplogroup has been observed in only one breed (Orino) in Southern Europe (Greece) also seems unusual [32]. The presence of haplogroup C in countries located around the Adriatic Sea is difficult to explain, taking into account that this haplogroup is also not observed even in Italian sheep breeds [32].

In order to compare the haplogroup frequencies in the Bulgarian sheep populations with those of other European populations, we performed a PCA analysis (Figure 3). The PCA plot revealed that all Bulgarian samples clustered close together with other sheep breeds from different European regions, which is due to the high frequency of haplogroup B in European sheep breeds. The outlier position of some European breeds may be associated with a relatively high frequency of haplogroup A (WAL and SHK) or the presence of rare haplogroups such as C (Cikta and BAR) and D (BOV) (Table S1). Also, the PCA plot clearly separated the Near Eastern sheep breeds (Turkey and Israel) from the main genetic pool due to the high frequency of haplogroup A compared to European breeds, as well as the presence of a relatively high percentage of haplogroup C (Figure 2). The Russian Native Sheep Breeds (RSB) also showed an outlier position since four haplogroups were observed: B (64.8%), A (28.9%), C (5.5%), and D (0.8%) (Table S1).

We constructed the NJ phylogenetic tree to demonstrate the phylogenetic relationship among studied sheep breeds (Figure 4). The phylogenetic analysis provides results largely in agreement with those obtained from the network analysis. The NJ phylogenetic tree formed two separate clades representing haplogroups B (more frequent) and A. Additional, phylogenetic tree revealed extensive haplotype sharing shows a weak genetic differentiation and the existence of gene flow between the breeds studied.

### 4.2. Genetic Differentiation and Similarities between Native Bulgarian Sheep Breeds

To analyze the genetic variation among and within breeds, we performed AMOVA and evolutionary divergence genetic distance analysis (Table 2 and Table 3). The AMOVA results indicated very low genetic variation between breeds (2.42%), while within breeds the genetic variability was rather high—97.58% (Table 3). These observations showed that the greatest part of the genetic diversity occurred among individuals. The estimates of evolutionary genetic distance between the eight native Bulgarian sheep breeds also showed very low values (Table 2). The lack of clear genetic differentiation between the native Bulgarian sheep breeds may be due to different reasons. The most probable cause for the low genetic structuration is the human selection pressure on the breeds. The observed gene flow between sheep breeds may be attributed to the efforts for optimal use of the production factors as well as for increasing the size of the obtained production. Modern selection in sheep breeding is related to the creation of highly productive animals and providing suitable conditions for the expression of their genetic predispositions [75,76]. According to the economically determined prospects, various local breeds of sheep formed and bred in precisely defined regions of the country are moved to other territories in order to increase the productivity of local flocks of animals. In this way, the genetic profile of the local breeds is lost, creating a prerequisite for genetic erosion and gradual erasure of their specifics and characteristics. The situation is further complicated by the introduction of high-yielding animals and the gradual replacement of local breeds, which negatively affects the national gene pool. An example of this is the presence of haplogroup A in Kotel and Patch-faced Maritza sheep, which is unusual, given the genetic profile of other studied sheep breeds (Table 2).

## 5. Conclusions

The present study analyzed the polymorphism of mtDNA (D-loop region) in eight native Bulgarian sheep breeds. The obtained results indicated significant genetic diversity in the studied populations, with 225 distinct haplotypes. The majority of samples showed a high prevalence of the European haplogroup B (95.2%) while the remaining individuals were assigned to haplogroup A (4.8%). None of the other known mitochondrial haplogroups were detected. The constructed median-joining network showed that almost all haplotypes formed joined clusters among the populations, which indicated very low genetic differentiation and possibly a gene flow between breeds. The obtained results from this study will be useful for improving strategies for the conservation of native Bulgarian sheep genetic resources. Continuing research on large samples from other native sheep breeds will provide a more complete pattern of the genetic diversity and demographic history of native Bulgarian sheep populations.

## Figures and Tables

**Table 1:** Number of haplotypes (h), nucleotide diversity (π), Haplotype diversity, (*H*_d_), Average number of nucleotide differences (*K*_t_), Number of segregating sites (S), Fu and Li’s D and F tests, and Tajima’s D in eight Bulgarian native sheep breeds.

Breed	No. of Sequences	h	π	*H*_d_	*K*_t_	S	Fu and Li’s D	Fu and Li’s F	Tajima’s D
BRE	17	11	0.009	0.912	10.471	45	−0.69502 *p* > 0.10	−0.79361 *p* > 0.10	−0.88686 *p* > 0.10
KAR	60	40	0.010	0.981	11.892	117	−1.23094 *p* > 0.10	−1.66000 *p* > 0.10	−1.83455 *p* < 0.05
KOT	36	31	0.016	0.995	18.705	112	−2.03216 *p* > 0.10	−2.29412 *p* < 0.05	−1.91532 *p* < 0.05
BHPL	32	27	0.011	0.986	12.617	97	−1.68405 *p* > 0.10	−1.89471 *p* > 0.10	−1.80146 *p* > 0.10
CRSH	34	28	0.011	0.986	12.617	105	−0.02132 *p* > 0.10	−0.47016 *p* > 0.10	−1.20019 *p* > 0.10
MRS	48	27	0.009	0.953	11.359	87	−0.82732 *p* > 0.10	−1.20424 *p* > 0.10	−1.49593 *p* > 0.10
LSTZ	69	54	0.013	0.991	15.014	147	−0.59446 *p* > 0.10	−1.20335 *p* > 0.10	−1.75938 *p* > 0.10
PFM	14	13	0.017	1.000	20.333	78	−0.32847 *p* > 0.10	−0.49186 *p* > 0.10	−0.86217 *p* > 0.10

Abbreviations: BRE—Breznishka sheep; KAR—Karakachan sheep; KOT—Kotel sheep; BHPL—Pleven blackheaded sheep; CRSH—Copper-Red Shumen sheep; MRS—Middle Rodopean sheep; LSTZ—Local Starozagorska sheep; PFM—Patch-faced Maritza.

**Table 2:** Estimates of evolutionary genetic distance between eight native Bulgarian sheep breeds (as generated by MEGA software).

Population.	KAR	PBH	CRSH	KOT	BRE	MRS	LZST	PFM
KAR	0.000							
PBH	0.01040	0.000						
CRSH	0.01323	0.01410	0.000					
KOT	0.01348	0.01442	0.01619	0.000				
BRE	0.00979	0.01038	0.01326	0.01352	0.000			
MRS	0.00992	0.01075	0.01352	0.01393	0.01002	0.000		
LZST	0.01120	0.01196	0.01456	0.01473	0.01125	0.01163	0.000	
PFM	0.01509	0.01587	0.01770	0.01776	0.01522	0.01541	0.01623	0.000

Abbreviations: BRE—Breznishka sheep; KAR—Karakachan sheep; KOT—Kotel sheep; BHPL—Pleven blackheaded sheep; CRSH—Copper-Red Shumen sheep; MRS—Middle Rodopean sheep; LSTZ—Local Starozagorska sheep; PFM—Patch-faced Maritza.

**Table 3:** Analysis of molecular variance for overall population’s genetic diversity.

Source of Variation	Degrees of Freedom d.f.	Sum of Squares, SS	Variance Components, VC	Percentage of Variation V %
Among populations	7	96.897	0.17608	2.42
Within populations	307	2180.386	7.10223	97.58
Total	314	2277.283	7.27832	

## Data Availability

The authors can provide the data if needed.

## Supplementary Materials

The following supporting information can be downloaded at: https://www.mdpi.com/article/10.3390/ani13233655/s1, Table S1: Source data for the PCA of Eurasian sheep beeds. Haplogroup frequecies are calculated on different haplotypes. Macro area, country of origin, breeds, and acronyms used in PCA analysis.; Table S2: Genetic differentiation based on haplotype frequencies among eight native Bulgarian sheep breeds.
