# Supplementary material for: Mitochondrial Diversity and Phylogenetic Relationship of Eight Native Bulgarian Sheep Breeds
Source: Animals (Basel). 2023 Nov 25;13(23):3655. doi: 10.3390/ani13233655 (PMC10705445; doi:10.3390/ani13233655)
Supplement: Supplementary file 1 [file animals-13-03655-s001.zip › Table S2.pdf]

Table S2. Genetic differentiation based on haplotype frequencies among eight native Bulgarian sheep breeds.

POPULATION 1	POPULATION 2	Fst
BRE	KAR	0.05559
BRE	KOT	0.05747
BRE	PBH	0.04620
BRE	CRSH	0.04240
BRE	MRS	0.06421
BRE	LSTZ	0.04201
BRE	PFM	0.04879
KAR	KOT	0.02468
KAR	PBH	0.02194
KAR	CRSH	0.01449
KAR	MRS	0.02544
KAR	LSTZ	0.00927
KAR	PFM	0.01262
KOT	PBH	0.05006
KOT	CRSH	0.00464
KOT	MRS	0.05349
KOT	LSTZ	0.01027
KOT	PFM	0.02275
PBH	CRSH	0.03202
PBH	MRS	0.03882
PBH	LSTZ	0.01739
PBH	PFM	0.03660
CRSH	MRS	0.02873
CRSH	LSTZ	0.00717
CRSH	PFM	0.01562
MRS	LSTZ	0.03624
MRS	PFM	0.03045
LSTZ	PFM	0.00270

Abbreviations: BRE – Breznishka sheep; KAR - Karakachan sheep; KOT – Kotel sheep; BHPL – Pleven blackheaded sheep; CRSH - Copper-Red Shumen sheep; MRS – Middle Rodopean sheep; LSTZ – Local Starozagorska sheep; PFM - Patch-faced Maritza
